# Solidification Cracking Assessment of LTT Filler Materials by Means of Varestraint Testing and µCT

**DOI:** 10.3390/ma13122726

**Published:** 2020-06-15

**Authors:** Florian Vollert, Maximilian Thomas, Arne Kromm, Jens Gibmeier

**Affiliations:** 1 Institute for Applied Materials (IAM-WK), Karlsruhe Institute of Technology (KIT), Engelbert-Arnold-Straße 4, 76131 Karlsruhe, Germany; jens.gibmeier@kit.edu; 2 Bundesanstalt für Materialforschung und -Prüfung (BAM), Unter den Eichen 87, 12205 Berlin, Germany; maximilian.thomas@bam.de (M.T.); arne.kromm@bam.de (A.K.)

**Keywords:** LTT weld filler materials, µCT-analysis, hot cracking, welding, varestraint test

## Abstract

Investigations of the weldability of metals often deal with hot cracking, as one of the most dreaded imperfections during weld fabrication. The hot cracking investigations presented in this paper were carried out as part of a study on the development of low transformation temperature (LTT) weld filler materials. These alloys allow to mitigate tensile residual stresses that usually arise during welding using conventional weld filler materials. By this means, higher fatigue strength and higher lifetimes of the weld can be achieved. However, LTT weld filler materials are for example, high-alloyed Cr/Ni steels that are susceptible to the formation of hot cracks. To assess hot cracking, we applied the standardized modified varestraint transvarestraint hot cracking test (MVT), which is well appropriate to evaluate different base or filler materials with regard to their hot cracking susceptibility. In order to consider the complete material volume for the assessment of hot cracking, we additionally applied microfocus X-ray computer tomography (µCT). It is shown that by a suitable selection of welding and MVT parameter the analysis of the complete 3D hot crack network can provide additional information with regard to the hot cracking model following Prokhorov. It is now possible to determine easy accessible substitute values (e.g., maximum crack depth) for the extent of the Brittleness Temperature Range (BTR) and the minimum critical strain $P_{min}$.

## 1. Introduction

The aim to achieve lightweight constructions and higher load capacities at the same time leads to an increased use of high-strength steels. To exploit their full strength potential, welding these components on their own strength level is a major challenge. Therefore, the residual stress state in the weld joint is of great importance, as it strongly affects the cold cracking risk and fatigue life of the welded components. Two main mechanisms usually lead to the final residual stress state after welding of ferritic steels, that is, the restraining effect and the martensite formation. Due to the cold surrounding base material, the thermal contraction during cooling down of the weld joint is restrained. Together with inhomogeneities of the temperature distribution, this effect leads to the formation of tensile residual stresses. The second effect is that the phase transformation austenite to martensite leads to an increase of weld volume, which is also hindered by the base material. This counteracts the restrained thermal shrinkage of the weld and shifts the residual stress distributions towards compression. Both mechanisms superimpose and lead to the resulting residual stress state, which is in tension if the restrained thermal shrinkage is dominating. To mitigate welding induced tensile residual stresses, post-weld treatments can be applied with the objective to increase the fatigue resistance, as for example, shot peening, hammering or heat treatments. However, these techniques are either time consuming or cost intensive. Hence, it is preferable to reduce tensile residual stresses in the weld line without post-weld treatments, that is, during the welding process. Using low transformation temperature (LTT) weld filler materials is an innovative method to mitigate welding tensile residual stresses directly during weld fabrication. Its effectiveness has been proven in numerous research projects (e.g., References [1,2,3,4,5]. Most of the works deal with the basic verification of the generated residual compressive stresses [6,7,8] and/or their influence on the fatigue strength [9,10,11]. Comprehensive overviews can be found from Ooi et al. [12] and Kromm et al. [2]. Recent studies have focused on extended topics such as microstructure, the associated mechanical properties [13,14] and the behaviour during multi-layer welding [15,16,17,18,19]. Compared to conventional weld filler materials, the martensite transformation is delayed, which is achieved by the addition of alloying elements like for example, nickel or manganese. The volume expansion during martensite formation is more pronounced at lower temperatures due to the higher coefficient of thermal expansion (CTE) for austenite compared to martensite. In-situ analysis using high-energy synchrotron X-ray diffraction during a realistic Metal Active Gas (MAG) welding process showed a significantly higher decrease of residual strain due to the hindered volume expansion for a LTT weld filler material compared to a conventional weld filler material [20,21]. The subsequent residual stress analysis using the contour method, which gives an entire two-dimensional residual stress map, revealed a higher compressive residual stress level for the investigated LTT filler materials compared to a conventional weld filler material [22].

However, since LTT alloys are high-alloyed filler materials (e.g., Cr/Ni steels) they may show high hot cracking susceptibilities depending on the chemical composition. This circumstance was extensively investigated by the authors in a previous study [23] using the MVT test. As the surface based MVT standard analysis approach did not produce clear trends and left some open questions, µCT-imaging was carried out in order to examine crack-afflicted areas below the specimen surface. This allowed for accurate quantification of cracking in each specimen, giving a consistent ranking of the examined alloys solidification cracking susceptibilities.

It became clear, though, that the µCT results from Reference [23] were not yet exploited to their full potential. The current study therefore aims to develop a method for detailed characterization of sub-surface crack networks with regard to the hot cracking model following Prokhorov. This generates valuable insights into the underlying mechanisms of solidification cracking depending on different process parameters.

## 2. State of the Art

Hot cracks are formed at high temperatures during the solidification of the weld pool or during reheating (e.g., multi-pass welding). They are intergranular or interdentritic defects since mostly low melting phases at the grain boundaries are involved during hot crack formation.

Dealing with hot cracking, at first an appropriate classification is required. In this regard, it is well accepted to distinguish between solidification cracks (SCs), liquation cracks (LCs) and ductility dip cracks (DDCs) [24]. Their formation is influenced by numerous factors and their complex interactions [25]. Basically, these factors can be differentiated into three main groups [26]:

- Material specific factors (e.g., chemical composition)
- Design specific factors (e.g., specimen geometry or restraint)
- Welding parameters (e.g., heat input)

The chemical composition particularly influences the solidification interval and the tendency to form low melting phases. As an example, it is known that Ni forms a low melting eutectic at 637 °C together with impurities (e.g., Sulphur) [27] which is probably one reason of the high hot cracking susceptibility of LTT weld filler materials. Additionally, the primary solidification behaviour significantly influences the hot cracking susceptibility of a material. Steels with a Ni-equivalent to Cr-equivalent ratio Creq/Nieq < 1.35 solidify primarily austenitic [28]. As the solubility and diffusion rate of elements like for example, sulphur that promote hot cracking is lower in the face cantered cubic (fcc) structure compared to the body cantered cubic (bcc) structure, primary austenitic solidification favours the formation of hot cracks [29]. Consequently, studies with varying chemical compositions of LTT weld filler materials revealed decreasing hot crack susceptibility with increasing $Cr_{eq}/Ni_{eq}$ ratio [23]. Important welding parameters with regard to hot cracking susceptibility are the arc voltage U, welding current I and the travel speed *v*${}_{w}$. These parameters can be summarized by the heat input per unit length *E*${}_{w}$:

(1) $$E_{w}=\frac{U\cdot I}{v_{w}}.$$

*E*${}_{w}$ is a measure of the thermal energy supply to the weld line. In particular, it influences the cooling rate. A rapid cooling leads to supercooled melt at the solidification front, which results in a dendritic crystal growth. These so called “mushy zones” favour low melting phases, because the balance between solid and liquid phase is not achieved in the interdentritic zones. With increasing solidification rates, the extension of the “mushy zones” and consequently the solidification cracking susceptibility increases [30]. Furthermore, numerous additional influencing factors are relevant during solidification and need to be considered as a whole to effectively describe the formation of solidification cracks. From a technical perspective, all this is difficult to consider and the existing theoretical models usually only consider particular aspects. However, basically two criteria must be fulfilled in a solidification crack critical temperature range that solidification cracks can arise [31]:

1. There has to be a solidification crack susceptible microstructure
2. Thermal or mechanical strains have to be present

Most theoretical approaches are based on one of these two criteria such as the Rate of Feeding/Rate of Shrinkage (ROF/ROS) model described by Feurer [32] which is a representative for criterion 1. A further important approach is the hot crack model following Prokhorov (criterion 2) that is briefly introduced in the following.

### 2.1. Hot Cracking Model Following Prokhorov

**Figure 1:**
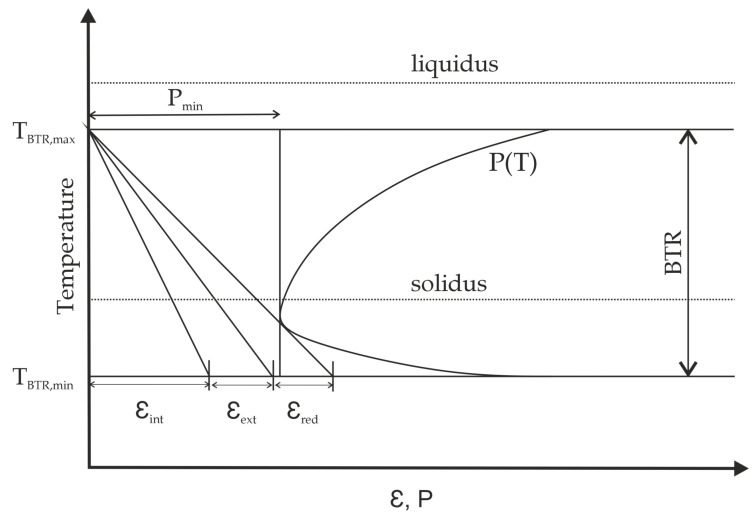
Hot cracking model following Prokhorov [34]. Hot cracks form if the total strain exceeds the critical strain function P(T) within the Brittleness Temperature Range (BTR).

Prokhorov described a hot cracking model with the assumption that a material specific hot cracking critical temperature range exists [33,34]. Following Prokhorov, this temperature interval is called “Brittleness Temperature Range” (BTR). The BTR starts below the liquidus temperature at $T_{BTR,max}$ and ends below the solidus temperature at $T_{BTR,min}$. Additionally, a critical strain needs to be exceeded for hot crack initiation. Hence, a temperature dependent critical strain function P(T) in the BTR is defined by Prokhorov. The acting strain in the material can either be externally ($\varepsilon_{ext}$) (e.g., mechanical load) or internally ($\varepsilon_{int}$) (e.g., restrained thermal shrinkage) induced. Hot cracking occurs if the total strain $\varepsilon_{tot}=\varepsilon_{ext}+\varepsilon_{int}$ exceeds P(T) within the BTR. Figure 1 schematically illustrates the hot cracking model following Prokhorov. At temperatures below $T_{BTR,min}$ the grain boundary strength is high enough to endure occurring strains. These two opposing mechanisms within the BTR explain the qualitative evolvement of a critical strain function P(T). At high temperatures within the BTR the hot crack formation is limited by the rate of feeding (ROF). Here, P(T) decreases until $P_{min}$ is reached. From here on the determining factor is the grain boundary strength and P(T) increases until $T_{BTR,min}$ is reached.

However, this fundamental model only examines a sub-aspect and there exist further-developed models that base on fundamental ones described above. For instance, the mechanisms that lead to the existence of the upper and lower temperature bounds $T_{BTR,max}$ and $T_{BTR,min}$ are not considered in the model according to Prokhorov. In addition, the qualitative evolvement of P(T) is not explained with regards to the microstructural processes that take place in the BTR. Using the term BTR one must also admit that also this naming is discussed controversially, but this more philosophical contemplation is out of the scope of our work. As most hot cracking tests exactly exploit the correlations described by Prokhorov (a critical load is applied in the BTR), for the understanding of our explanation of solidification cracking in Cr/Ni LTT alloys the introduction given before is adequate.

### 2.2. Modified Varestraint Transvarestraint (MVT) Hot Cracking Test

**Figure 2:**
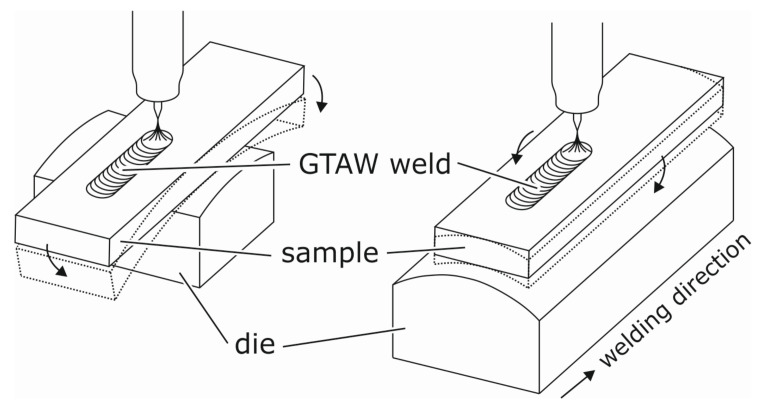
The modified Varestraint-(left) and Transvarestraint (right) Test [23].

One of the most commonly used hot cracking tests is the Modified Varestraint Transvarestraint (MVT) test (Figure 2) which is described in ISO TR 17641-3 [35]. In case of testing filler materials, at first, a U-shaped groove with a depth of 5 mm and a width of 20 mm is milled in longitudinal direction into a blank specimen and the investigated weld filler material is deposited into the groove by for example, automated gas metal arc welding using several subsequently deposited weld beads. Afterwards, the specimen is cut to the standardized MVT dimensions (100 mm × 40 mm × 10 mm). The MVT test is performed using an automated gas tungsten arc welding (GTAW) process with defined heat input. As soon as the weld pool reaches the specimen centre, the specimen is bent over a die with defined radius either in longitudinal (Varestraint) or transverse (Transvarestraint) strain direction. By this means, a defined bending rate is applied. The bending strain is varied using different die radii $r_{d}$. The total strain $\varepsilon_{tot}$ at the specimen surface can be calculated according to:

(2) $$\varepsilon_{tot}\approx \frac{100\cdot h}{2\cdot r_{d}},$$

with *h* = 10 mm as specimen thickness. Hot cracks are formed locally behind the weld pool wherever the applied strain exceeds the critical strain P(T) within the critical temperature range BTR. If $\varepsilon_{tot}$ is sufficiently large the crack length corresponds to the distance of the isotherms $T_{BTR,max}$ and $T_{BTR,min}$. According to ISO TR 17641-3 the evaluation is carried out using an optical light microscope at a magnification of 25×.

In the Varestraint test, the cumulated crack length is usually determined. By this means, both SCs and LCs in the weld metal and in the heat affected zone are evaluated. Then, the investigated weld filler material is ranked in regard to its hot cracking susceptibility [36]. Because of the directionality of the strain distribution in the Transvarestraint test it has a high sensitivity to solidification cracking and a centreline crack is favored in the WM. Consequently, it is sufficient to consider only the maximum crack length (usually of the centreline crack) during evaluation. This should lead to similar rankings of the investigated materials compared to an evaluation, where the cumulated crack length would be determined [31].

As a result, information about the extension of the hot cracking susceptible temperature range BTR can be extracted from standard MVT test by using the easily accessible substitute value of maximum crack length. However, the standard evaluation only considers surface information. Hence, for accurate assessment of the hot cracking susceptibility of a weld filler material, information from the material volume should also be considered in addition. To realize this, we enhanced the standard evaluation by using microfocus X-ray computer tomography (µCT), which allows determining and evaluating the entire 3D hot crack network of a MVT specimen.

**Figure 3:**
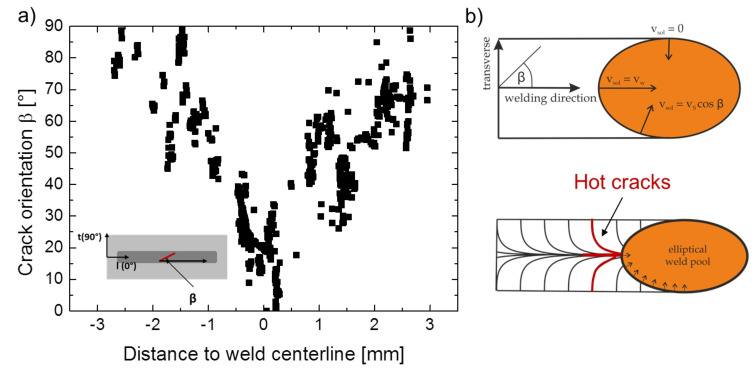
Crack orientation as a function of the distance to the weld centreline (**a**). Crystallization direction for an elliptical weld pool [31] (**b**). Apparently, the crack orientation corresponds to the crystallization direction [37].

Using this approach also the crack orientation can be evaluated. In Figure 3a as an example the crack orientation determined this way for the MVT specimen with a travel speed of $v_{w}$ = 3 mm/s is shown [37]. As hot cracks preferabily arise along high angle grain boundaries [38], the graph illustrates a clear dependency of crack orientation and solidification direction during cooling down of the weld. In Figure 3b, the schematic illustration of the solidification process for an elliptical weld pool is presented. The solidification rate $v_{sol}$ and the crystallization direction are orthogonal to the liquid-solid interface of the weld pool. Consequently, starting at an angle β of 90° to the longitudinal axis, β progressively decreases upon moving to the centreline. At the centreline the two opposing solidification fronts impinge on each other, resulting in a macroscopic centreline grain boundary with an orientation of β = 0°. The solidification direction varies with the travel speed as the weld pool shape is significantly influenced. With increasing travel speed, the shape evolves from an elliptical to a teardrop shape [31].

Furthermore, as described in Reference [37] the maximum crack depth turned out to be one crucial parameter with regard to hot cracking susceptibility. Like the 3D temperature distribution, the 3D strain distribution during bending is not accessible without great effort. However, as the strain decreases with increasing distance to the weld surface, the maximum crack depth $D_{max}$ of the 3D hot crack network should be proportional to the minimum critical strain Pmin. As a result, $D_{max}$ acts as accessible substitute value and it is possible to obtain rankings with regard to $P_{min}$ [37]. This evaluation approach is promising to compare the overall hot crack susceptibility of different weld filler materials. Hence, in this study we applied this new approach of data recording and data treatment to explain the hot cracking mechanism on basis of 3D-data of the weld crack network, that is, µCT is used in this study to determine the entire 3D hot crack network of MVT specimens welded with a Cr/Ni based LTT weld filler material and different travel speeds $v_{w}$. Subsequently, the reconstructed images from the µCT scans are analysed.

Derived from the crack orientation, the 3D weld pool is reconstructed to gain information about the weld pool geometry during the MVT test. Moreover, in order to evaluate the extent of the brittleness temperature range BTR, the 3D isotherms $T_{BTR,min}$ and $T_{BTR,max}$ are determined by performing ellipse fitting. Based on these results, the mechanism, dependent on the travel speed that lead to the formation of the 3D hot crack network during MVT test, are discussed.

Moreover, as the welding and MVT parameters are usually chosen empirically. It can be observed that the hot cracking susceptibilities of different materials are influenced by the testing conditions in very different, sometimes controversial ways. As an example, increasing travel speed was reported to either increase [39] or decrease [40] the hot cracking susceptibility. This deviant behaviour for different materials can be critical, as the chosen welding parameters can alter the materials ranking with regard to hot cracking susceptibility. We intend to address the question whether the information about the evolving 3D crack network can be used to find testing parameters that allow for a better comparability of materials in regard to their hot cracking susceptibility.

## 3. Experimental Method

### 3.1. Materials and MVT-Testing

We focused our investigation on a Cr/Ni based LTT alloy (e.g., References [1,41]) that shows a conspicuous hot cracking characteristic [23]. The investigated samples were standardized MVT specimens with dimensions 100 mm × 40 mm × 10 mm. The filler material was deposited into the groove of the MVT specimen by automated gas metal arc (GMAW) welding using six layers while the low alloyed high strength steel S960Q was used as substrate. The chemical compositions of the LTT filler material and the base material are listed in Table 1.

After welding, the specimens were finished to the standardized MVT dimensions by milling excess weld metal. During MVT testing, the LTT welds were re-melted by automated GTAW-welding using different heat inputs between 6.0–15 kJ/cm. Main influence on the heat input was the travel speed of the welding torch, which was varied between 1.8 mm/s and 3 mm/s. The second influence parameter was the welding current, which was varied between 180 A and 210 A (see Table 2). Bending of the MVT specimens was executed during welding in longitudinal direction to the weld line (Varestraint-modus) using a bending radius of 125 mm (resulting surface strain of 4%). An overview of the welding and MVT parameters can be found in Table 2.

### 3.2. µCT Analysis

µCT was applied using the High-Resolution Cone-Beam CT system type Yxlon Y.CT Precision with fine focus twin head FXE 225.99. The scans were performed at a high voltage of 190 kV and a current of 0.3 mA. The detailed Tube- and Scanparameter are listed in Table 3.

**Figure 4:**
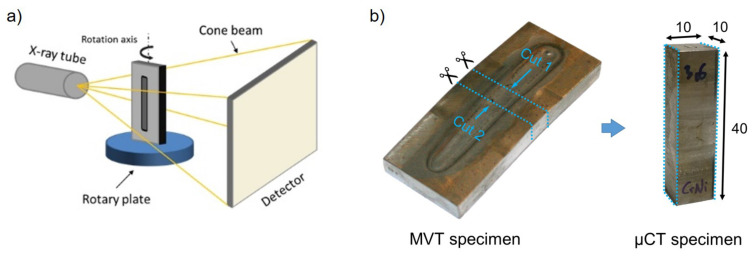
Schematic illustration of the microfocus X-ray computer tomography (µCT)-scan of the whole specimen to obtain the position of the hot crack network [44] (**a**). Whole MVT specimen and cutted µCT specimen (**b**).

To determine the location of the hot crack network, the whole MVT specimen was scanned (Figure 4a). Subsequently, to reduce X-ray absorption and to increase the resolution of the reconstructed images, the investigated specimen was cut into a smaller cuboid (Figure 4b). Because of the cone beam used in lab CT analysis the image resolution becomes better the closer the specimen can be positioned to the X-ray tube. In our experiment, this resulted in a voxel size of the cut specimen of about 7 µm and in case of the uncut specimen about 25 µm. In the next step, the obtained reconstructed images were segmented, which means that each voxel is either assigned to “crack” or “no crack”. A two-step segmentation strategy was implemented using MATLAB©. In the first step, an adaptive thresholding algorithm (described in Reference [42]) was applied. Afterwards, the segmented voxels from the first step are used as “seeds” for a region-growing algorithm [43]. More detailed information about the segmentation approach can be found in Reference [37]. Subsequently, the segmented images were statistically and quantitatively analysed.

## 4. Data Analysis and Results

### 4.1. Weld Pool Reconstruction

**Figure 5:**
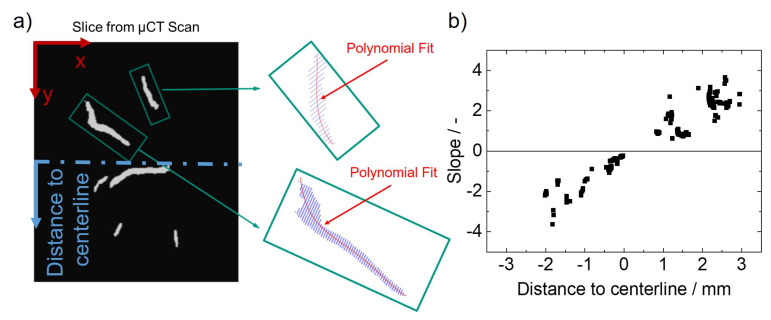
Schematic illustration of the data processing for one individual slice from the segmented 3D information from the µCT analysis. A polynomial of degree 2 is fitted to each individual crack (**a**). The derivative provides the crack slopes as a function of the distance to the weld centreline (**b**).

The crack orientation was evaluated by performing a polynomial fit of degree 2 on each individual crack in each µCT slice (Figure 5a). The derivatives of the fitted polynomials provide the slopes of the cracks (Figure 5b). Consequently, the slopes describe the crystallization direction as it is assumed that the cracks are formed along the temperature gradient. The crystallization direction is orthogonal to the liquid-solid interface of the weld pool. Under the assumption of an elliptical weld pool with the common ellipse equation:

(3) $$y=\frac{b}{a}\sqrt{a^{2}-x^{2}}.$$

The derivative d*x*/d*y* of Equation (3) can be fitted on the rotated slope of the crystallization direction giving the semi axis of the weld pool length *a* and the weld pool width *b*.

**Figure 6:**
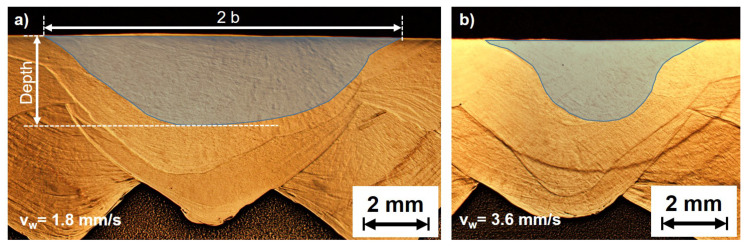
Determination of the weld pool depth from metallographic cross-sections, exemplarily for the travel speed $v_{w}=1.8$ mm/s (**a**) and $v_{w}=3.6$ mm/s (**b**).

Figure 6 exemplarily shows metallographic cross-sections for the specimens welded with a travel speed $v_{w}=1.8$ mm/s (a) and $v_{w}=3.6$ mm/s (b) to determine the weld pool depths.

### 4.2. Determination of the Isotherms $T_{BTR,min}$ and $T_{BTR,max}$

**Figure 7:**
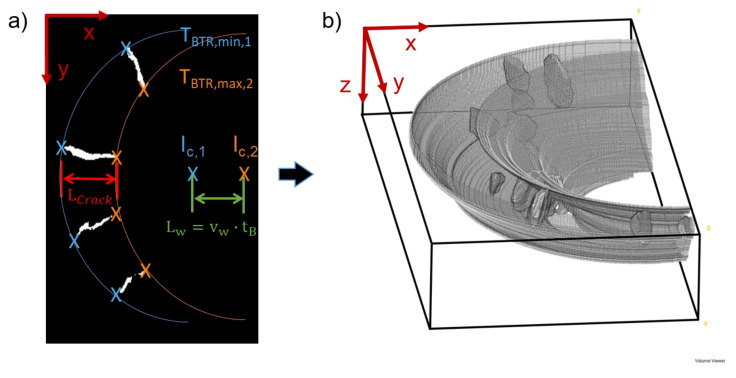
Exemplarily determination of the isotherms $T_{BTR,min}$ and $T_{BTR,max}$ for one µCT slice (**a**). 3D illustration of the determined Brittleness Temperature Range (BTR) (**b**).

Following the approach of Prokhorov, hot cracks arise between the isotherms of $T_{BTR,min}$ and $T_{BTR,max}$. However, it must be taken into account that during the bending process, the BTR moves along the longitudinal axis in welding direction by the length $L_{w}=v_{w}\cdot t_{B}$ (with $t_{B}$ as bending time) (Figure 7a). As a result, the maximum crack length is limited by the distance of the isotherm $T_{BTR,min,1}$ and $T_{BTR,max,1}$ at the starting time of bending and the added length $L_{w}$ as distance between the isotherm $T_{BTR,max,1}$ (start of bending) and $T_{BTR,max,2}$ (end of bending). Ellipse fitting (assumption of elliptical weld pool) with $T_{BTR,min,1}$(blue crosses in Figure 7a), $T_{BTR,max,2}$ (orange crosses in Figure 7a) of each individual crack and the weld pool centres $l_{c,1}$/$l_{c,2}$ as input data can be performed for each individual µCT slice. This approach provides depth information about the 3D course of the crack length $L_{Crack}$ as distance between the two fitted ellipsoids at the weld centreline and thus about the extent of the BTR (Figure 7b). This procedure was applied for all process variations investigated in this work.

### 4.3. Application on MVT test

**Figure 8:**
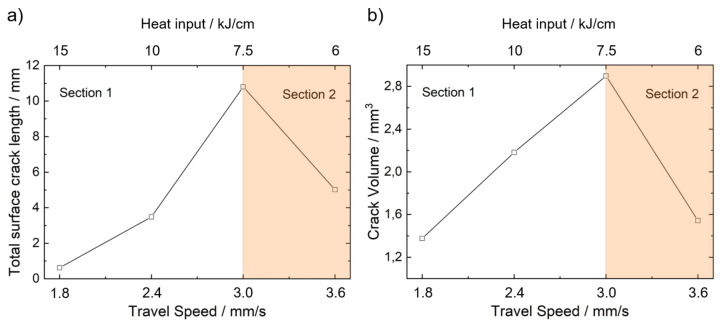
Total surface crack length as function of travel speed (determined by standard MVT evaluation) (**a**). Total crack volume as a function of travel speed (determined by µCT) (**b**).

For the investigated LTT weld filler material, Figure 8 shows the influence of travel speed on the determined total cumulated surface crack length (a) and the total cumulated crack volume (b). Both curves can be divided into two parts. Between 1.8 mm/s $\leq v_{w}\leq$ 3.0 mm/s the crack volume increases with increasing travel speed (Section 1). After the maximum value at $v_{w}=3$ mm/s is reached, the crack volume decreases by further increasing of the travel speed (Section 2). Obviously, increasing the travel speed can either increase (low travel speed) or decrease (high travel speed) the extent of hot cracking during the MVT test.

**Figure 9:**
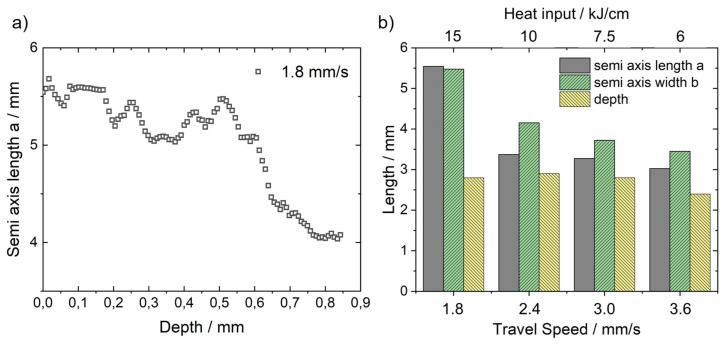
Semi axis length a of the reconstructed elliptical weld pool for $v_{w}=1.8$ mm/s as a function of depth (**a**). Semi axis length a, width b of the elliptical weld pool and depth (metallographic analysis) as a function of the travel speed (**b**).

To examine the mechanisms that lead to this behaviour, the weld pool geometry was reconstructed from the crack orientations. Exemplarily for $v_{w}=1.8$ mm/s, the depth profile of the semi axis length a of the ellipse fitted to the isotherms is plotted in Figure 9a. The length remains approximately constant up to a depth of about 0.5 mm. Then, a steady decline of the length can be observed up to a depth of about 0.85 mm. It is notable that weld pool reconstruction can only be performed up to a depth were sufficient cracks are present across the entire weld width. If this is no longer possible, the calculation is aborted. In the case of $v_{w}=1.8$ mm/s the maximum depth was about 0.85 mm.

The determined semi axes of the weld pool length and width of the weld surface are shown in Figure 9b for varying travel speeds. Additionally, the weld pool depth, determined from metallographic cross-sections are also given. Because of the decreasing heat input with increasing travel speed the weld pool volume decreases. Consequently, the amount of material at temperatures within the BTR during bending also decreases. This should lead to a reduction of the hot crack volume if effects due to the different cooling conditions are neglected. However, between 1.8 mm/s $\leq v_{w}\leq$ 3.0 mm/s the crack volume increases even though the weld pool is getting smaller.

**Figure 10:**
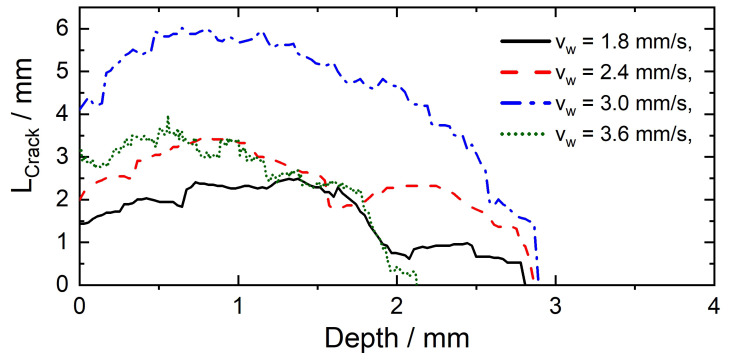
Distance $L_{Crack}$ between the two fitted ellipses for MVT testing in Varestraint mode of Cr8Ni6 LTT weld filler material.

Figure 10 shows the distance $L_{Crack}$ as a function of depth, determined by ellipse fitting for the investigated travel speeds. Generally, the depth curve for all travel speeds initially increases to a maximum value underneath the weld surface. This observation is consistent with the depth courses of the cumulated crack length shown for example, in References [23,45]. Even though the highest bending strains act at the surface, the graphs reveal a maximum total crack length below the surface. Kannengiesser et al. [45] attributed this observation to the directed solidification kinetics in the material volume, which is responsible for the high hot cracking susceptibility beneath the weld surface. From $v_{w}=1.8$ mm/s to 3.0 mm/s the maximum value for $L_{Crack}$ increases. However, at $v_{w}=3.6$ mm/s $L_{Crack,max}$ is smaller (approximately 4 mm) compared to $v_{w}=3.0$ mm/s ($L_{Crack,max}\approx 6$ mm). The maximum crack depth $D_{max}$ is approximately constant for $v_{w}=1.8$ mm/s to 3.0 mm/s ($D_{max}\approx 3$ mm) and is 2.1 mm for $v_{w}=3.6$ mm/s.

In the following discussion of the results, we want to explore why there obviously exist two sections with contrary trends regarding the hot cracking behaviour. Furthermore, we will pursue the issue whether the additional information from the weld volume provided by µCT analysis can be used for the deduction of the underlying hot cracking mechanisms. In this context, we further address the question of whether the results will support to identify appropriate MVT testing parameters that allow for the better comparability of weld filler materials in regard to their hot cracking susceptibility. However, it must be noted that microstructural mechanisms (e.g., segregation) that result from the different solidification conditions are neglected in the following, as they are not necessarily needed for an understanding of the discussed mechanisms.

## 5. Discussion

### 5.1. Crack Length $L_{Crack}$

**Figure 11:**
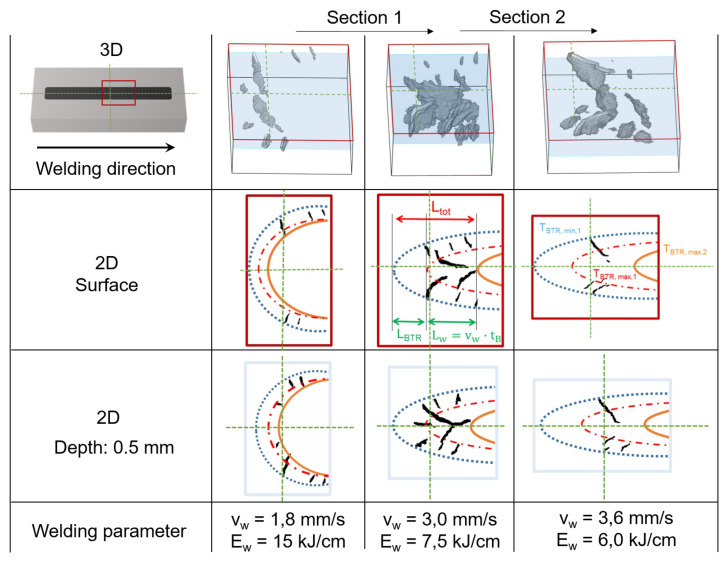
Schematic illustration of the isotherms $T_{BTR,min,1}$, $T_{BTR,max,1}$ (start of bending) and $T_{BTR,max,2}$ (end of bending), exemplarily for the surface and 0.5 mm depth slices of the µCT scans for three different travel speeds and for MVT testing in Varestraint mode.

As an example, Figure 11 schematically shows the segmented slices for the weld surface and in a depth of 0.5 mm determined by means of µCT for three different travel speeds. Crack initiation occurs preferentially near the fusion boundary, as here the orientation of the crystallization direction is advantageous with regard to the formation of hot cracks (orientation orthogonal to load direction). The maximum obtainable crack length consists of two length components. $L_{BTR}$ as the distance between the isotherms $T_{BTR,min,1}$ and $T_{BTR,max,1}$ (start of bending) and $L_{w}$ as the distance which the isotherms are shifted (moving torch) during the bending process. The sum of $L_{BTR}$ and $L_{w}$ gives the total length $L_{tot}$ that is displayed in Figure 11 and is equal to $L_{Crack}$ for travelling speeds from $v_{w}=1.8$ mm/s to 3.0 mm/s. With increasing travel speed, the distance $L_{w}$ increases. As a result, cracks can grow longer along the solidification direction towards the centreline. Consequently, the determined crack lengths $L_{Crack}$ increase from $v_{w}=1.8$ mm/s to 3.0 mm/s as shown in Figure 11. However, at $v_{w}=3.6$ mm/s it appears that the maximum possible crack length $L_{tot}$ with regard to the distance between the isotherms $T_{BTR,min,1}$ and $T_{BTR,max,2}$ is no longer obtained. As a result, $L_{Crack}$ is smaller than $L_{tot}$ because crack growing stops in the area of the centreline. Crack growing along the centreline grain boundary does not occur, as the loading direction in the Varestraint mode does not favour centreline cracking in longitudinal direction. Moreover, as can be seen in Figure 11, for $v_{w}=3.6$ mm/s not even the centreline is reached. One possible explanation for this could be that at higher travel speeds globular solidification may occur at the weld centre, which prevents hot cracking at the centreline as low melting phases, are distributed over a large area [46]. Consequently, $L_{Crack}$ of $v_{w}=3.6$ mm/s is smaller compared to $v_{w}=3.0$ mm/s (Figure 10). As a result, in order to compare different weld filler materials with regard to their BTR the travel speeds must be selected in such a way that the isotherm $T_{BTR,max,2}$ is the limiting factor for the crack length. If this is not the case (e.g., here for $v_{w}=3.6$ mm/s) there is a risk that comparing the crack lengths of different weld filler materials leads to erroneous rankings with regard to their hot cracking susceptibility.

### 5.2. Crack Depth $D_{max}$

**Figure 12:**
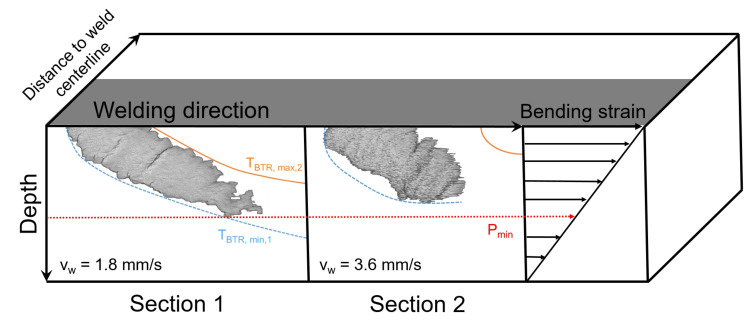
Schematic illustration of the limiting factors of hot crack formation in the two sections. Section 1: The crack length is limited by $T_{BTR,min,1}$ and $T_{BTR,max,2}$. The crack depth is limited by $P_{min}$. Section 2: The crack length is limited by the weld centreline and the crack depth by $T_{BTR,min,1}$.

The maximum crack depth $D_{max}$ is either limited by the depth profile of $T_{BTR,min}$ or the minimum critical strain $P_{min}$, according to Prokhorov. If $T_{BTR,min,1}$ is at greater depths than $P_{min}$, the maximum depth $D_{max}$ is limited by $P_{min}$ and should be approximately constant and independent from the chosen travel speed. This is the case for $v_{w}$ = 1.8–3.0 mm/s and is schematically shown in Figure 12 (Section 1) for $v_{w}=1.8$ mm/s. In Section 2 ($v_{w}=3.6$ mm/s), $T_{BTR,min}$ is the limiting factor of $D_{max}$. In this case, $D_{max}$ decreases with increasing travel speed. As a result, in order to use $D_{max}$ as substitute value for $P_{min}$, as described in Reference [37], sufficient heat input during welding must be ensured and the applied bending strain must not be too high, so that $T_{BTR,min}$ is situated at greater depths than $P_{min}$ (Section 1).

### 5.3. Crack Volume

Table 4 is giving the overview of the determined maximal values of $L_{Crack}$ (determined by ellipse fitting) $L_{Crack,max}$, the calculated length $L_{w}$, the maximum determined crack depth $D_{max}$ and the calculated minimum critical strain $P_{min}$ for the different travel speeds.

As a result, the course of the total crack volume as function of travel speed can be explained with two competing mechanisms:

- Increasing travel speed results in an increasing length $L_{w}$ because $T_{BTR,max,2}$ moves during the bending process. Hence, the distance between $T_{BTR,min,1}$ and $T_{BTR,max,2}$ increases and longer cracks can arise. However, it seems that the maximum crack length can also be limited by the centreline grain boundary as the loading direction (varestraint) does not favor perpendicular crack formation. If this is the case, further crack growing can no longer occur with increasing travel speed.
- With increasing travel speed the heat input is decreasing. Consequently, because of the lower depth of the temperature field, $D_{max}$ is limited by $T_{BTR,min}$.

First, at low travel speed and high heat input (Section 1) the crack length $L_{Crack}$ is limited by $T_{BTR,max,2}$ and the maximum depth $D_{max}$ is limited by $P_{min}$. As a result, with increasing $v_{w}$, the total crack volume increases because $L_{total}$ increases while $D_{max}$ is approximately constant. After the maximum crack volume is reached, the crack volume decreases (Section 2) and the crack length $L_{Crack}$ no longer corresponds to the distance between the isotherms $T_{BTR,min,1}$ and $T_{BTR,max,2}$. Instead, the maximum crack length is limited by the centreline of the weld joint. At the same time, $D_{max}$ decreases as it is now limited by $T_{BTR,min}$, which extends to lower depths with higher traveling speeds.

## 6. Conclusions

In the scope of the development of LTT weld filler materials, the externally loaded Modified Varestraint Transvarestraint (MVT) test was applied to investigate the solidification cracking susceptibility. The investigations were performed on a Cr/Ni LTT weld filler material (8 wt.% Cr and 6 wt.% Ni) using the varestraint mode and different travel speeds, respectively welding heat inputs. Unlike the standard MVT evaluation that only considers surface information, in this study a new µCT approach was applied to analyse the complete 3D hot crack network. This provides valuable additional information from the bulk of the MVT test with regard to the hot cracking model following Prokhorov. The following conclusions can be drawn:

- From the crack orientation determined from the µCT volume information the weld pools can be reconstructed.
- Ellipse fitting of planar sections through the crack network allows for the determination of the isotherms of the maximum temperature $T_{BTR,max}$ and minimum temperature $T_{BTR,min}$ of the Brittleness Temperature Range (BTR) according to the hot cracking model following Prokhorov.
- However, for the ellipse fitting to determine the BTR the movement of the welding torch during bending must be taken into account and its travel speed must be adjusted that the isotherm $T_{BTR,max,2}$ is certainly the limiting factor for the crack length. If this is not the case, there is a risk that comparing the crack lengths of different weld filler materials leads to erroneous rankings with regard to their hot crack susceptibility.
- Using this new approach, the distance $L_{Crack}$ between the isotherms $T_{BTR,min,1}$ and $T_{BTR,max,2}$ can be used as a measure for the extent of the BTR, which reflects a new experimental accessible MVT parameter according to the Prokhorov model.
- An additional parameter obtained from the µCT analysis is the maximum crack depth $D_{max}$, which can represent a substitute value for the minimum critical strain $P_{min}$.
- In this regard, it is important to ensure a sufficiently high heat input and a not too high bending strain that $P_{min}$ is certainly the limiting factor for $D_{max}$. In practice, this could be ensured by a $D_{max}$ that is smaller than the weld pool depth.

## Figures and Tables

**Table 1:** Chemical composition in wt% of the pure low transformation temperature (LTT) alloy and the base material determined by spectral analysis.

Material	Chemical Composition in wt.%							
	C	Cr	Ni	Si	Mn	Mo	V	Fe
LTT weld (Cr/Ni)	0.045	8.0	6.0	-	0.5	-	-	bal.
S960Q (base material)	0.18	0.8	2.0	0.5	1.6	0.6	0.1	bal.

**Table 2:** Welding and Modified Varestraint Transvarestraint (MVT) Parameters.

Welding Parameters				
Voltage/V	12			
Current/A	210	200	190	180
Travel speed/mm/s	1.8	2.4	3.0	3.6
Heat input/kJ/cm	15	10	7.5	6.0
**MVT Parameters**				
Stroke rate/mm/s	2			
Surface strain/%	4			

**Table 3:** Tube- and Scanparameter for the MVT specimens.

Tubeparameter (Reflection Tube)	
Target	Wolfram
Voltage	190 kV
Current	0.3 mA
**Scanparameter**	
Number of Projections	2700
Integration time	300 ms

**Table 4:** $L_{Crack,max}$, $L_{w}$, $D_{max}$, and calculated $P_{min}$ for the different travel speeds.

Travel Speed $v_{w}$ / mm/s	Heat Input $E_{w}$ / kJ/cm	$L_{\mathrm{Crack},\max}$ / mm	$L_{w}$ / mm	$D_{\max}$ / mm	$P_{\min}$ / %
1.8	15	2.46	0.81	2.81	1.75
2.4	10	3.46	1.08	2.89	1.69
3.0	7.5	6.02	1.35	2.89	1.69
3.6	6	3.97	1.62	2.1	2.32
